# The Poisons in Tobacco Smoke

**Published:** 1883-10

**Authors:** 


### The Poisons in Tobacco Smoke.

Herr Kissling of Bremen has published a useful paper on the poisonous constituents of tobacco smoke, among which he specifies as strong in quality, carbonic oxide, sulphurated hydrogen, prussic acid, picoline basis, and nicotine. The first three substances, however, occur in such small proportions, and their volatility is so great, that their share in the action of tobacco smoke on the system may be neglected. The picoline bases, too, are present in comparatively small quantity; so that the poisonous character of the smoke may be almost exclusively attributed to the large proportion of nicotine present. Only a small part of the nicotine in a cigar is destroyed by the process of smoking, and a relatively large proportion passes off with the smoke. The proportion of nicotine in the smoke depends, of course, essentially on the kind of tobacco; but the relative amount of nicotine which passes from the cigar into smoke depends chiefly on how far the cigar has been smoked, as the nicotine-contents of the unsmoked part of a cigar is in inverse ratio to the size of this part—that is, more nicotine the shorter the part. Evidently in a burning cigar, the slowly advancing zone of glow drives before it the distillable matters so that in the yet unburned portion a constant accumulation of them takes place. More relatively of this substance passes into smoke in the case of cigars that are poor in nicotine, than in the case of cigars with much of that substance. Nicotine, notwithstanding its high boiling point, has remarkable volatility.—*Med. Gazette, N. Y.*

